# Researcher–researched relationship in qualitative research: Shifts in positions and researcher vulnerability

**DOI:** 10.3402/qhw.v11.30996

**Published:** 2016-06-14

**Authors:** Målfrid Råheim, Liv Heide Magnussen, Ragnhild Johanne Tveit Sekse, Åshild Lunde, Torild Jacobsen, Astrid Blystad

**Affiliations:** 1 Department of Global Public Health and Primary Care, University of Bergen, Bergen, Norway; 2 Department of Occupational Therapy, Physiotherapy and Radiography, Bergen University College, Bergen, Norway; 3 Department of Obstetrics and Gynecology, Haukaland University Hospital, Bergen, Norway; 4 Department of Clinical Science, University of Bergen, Bergen, Norway; 5 Haraldsplass Deaconess University College, Bergen, Norway; 6 DIALOG, Bergen, Norway

**Keywords:** Researcher–researched relationship, reflexivity, qualitative research, researcher vulnerability, ethics

## Abstract

**Background:**

The researcher role is highly debated in qualitative research. This article concerns the researcher-researched relationship.

**Methods:**

A group of health science researchers anchored in various qualitative research traditions gathered in reflective group discussions over a period of two years.

**Results:**

Efforts to establish an anti-authoritarian relationship between researcher and researched, negotiation of who actually “rules” the research agenda, and experiences of shifts in “inferior” and “superior” knowledge positions emerged as central and intertwined themes throughout the discussions. The dual role as both insider and outsider, characteristic of qualitative approaches, seemed to lead to power relations and researcher vulnerability which manifested in tangible ways.

**Conclusion:**

Shifting positions and vulnerability surfaced in various ways in the projects. They nonetheless indicated a number of similar experiences which can shed light on the researcher-researched relationship. These issues could benefit from further discussion in the qualitative health research literature.

This article begins and ends in the reflexive turn of qualitative research (Altheide & Johnsen, 1994). Reflexivity concerns thoughtful, analytic self-awareness of researchers’ experiences, reasoning, and overall impact throughout the research process. Pre-understanding and openness, closeness and distance, the co-construction and situating of knowledge, trustworthiness and integrity, power relations, and ethical dilemmas are given primacy in the qualitative methodology (Dahlberg, Dahlberg, & Nyström, 2008; Finley, 2002; Gergen & Gergen, 2000; Kvale & Brinkmann, 2009; Lincoln & Guba, 1985). In this article, we reflect on the role of the researcher in the process of knowledge production as it emerged in a series of reflective group discussions between researchers based in the health sciences. Focusing on our own research experiences, our aim was to explore systematically our experience of fluctuations in “superior” and “inferior” knowledge positions and the related researcher vulnerability that emerged.

The relation between researcher(s) and researched has been a recurrent concern in the methodology literature. The privileged position of the researcher *vis à vis* the researched has been strongly emphasized. The inherent power imbalance between the parties and the ethical concerns pertaining to this imbalance are commonly dwelled upon, with particular attention to the predetermined asymmetric roles between the researcher and the researched. However, the literature simultaneously emphasizes that qualitative traditions all have “…a common epistemological ground: the researcher determination to minimize the distance and separateness of researcher-participant relationships,” as phrased by Karnieli-Miller, Strier, and Pessach (2009, p. 279). Furthermore, it is argued that defining what knowledge is to count in a concrete researcher–researched encounter is not necessarily the sole privilege of the researcher because participants bring their own agenda to the research situation (Karnieli-Miller et al., 2009). In the ethnographic literature, much attention is paid to the complexity of the role of the researcher as observer, as well as the contextual understanding of potentially opposing perspectives between the researcher and researched (Adler & Adler, 2002; Angrosino & Mays De Pérez, 2000; Hammersley & Atkinson, 1983/1992; Vitus, 2008). Role conflict related to being both an insider and an outsider, and the experience of resistance from the research participant are related themes that seem to call for further nuancing of the representations of inherent asymmetric relations (see, for instance, Burns, Fenwick, Schmied, & Sheenan, 2012; Dwyer & Buckle, 2009; Jack, 2008; Lalor, Begley, & Devane, 2006; Lee, 1993; Malacrida, 2007).

The *insider-outsider perspectives* are not new, but have been hotly debated for decades (see for example, Emerson & Pollner, 1988; Garfinkel, 1984; Lynch & Woolgar, 1988; Pollner & Emerson, 2001). The debates revolve around researcher positionality, what it means to be an insider or outsider in a given study setting, and how the researcher's status is negotiated throughout the research processes. Laura Nader (1969/1972) launched the dichotomy of *studying up/studying down* pertaining to researcher positionality in her classical work, holding that studying up contributes in vital ways to an understanding of the processes by which power and responsibility are exercised. Beyond informing our understanding of patterns of distribution, value, and power, Nader's call for studying up has posed new questions pertaining to the research relationship, and has been widely drawn upon. The researcher “studying up” may experience him- or herself moving into a research field of less “control” or “power,” so the approach calls for new reflections on the issues of access, methodology, attitudes, and ethics (Nader, 1969/1972, p. 301). Reflections on studying up or down may enhance the understanding of researcher experiences in our article.

We should emphasize that we fundamentally acknowledge the existence of an inherent imbalance in the relation between the researcher(s) and the researched in qualitative health research. Despite this, we will make a modest attempt to add to the debate about whether the researcher is by definition located in a privileged and superior position vis-à-vis the research subjects. Our aim is to use examples from our own research projects to reveal shifts in “superior” and “inferior” positions in researcher–researched relationships, in which ethical dilemmas and vulnerability surface on the part of the researcher. A further aim is to explore whether experiences from projects based in different qualitative traditions can shed additional light on the researcher–researched relationship.

## Reflective group discussions

The authors of this article are senior researchers (two professors, four associate professors), all women, who gathered in six reflexive group discussions over a period of 2 years. The group participants had backgrounds in nursing (two), physiotherapy (two), genetic counselling (one), and in acting/drama as a pedagogical tool (one). One of the nurses also held a PhD in social anthropology. They were colleagues in research and/or in the running of a master's programme in health science. The participants reflected on their role as researchers in their earlier research projects. The reflective group discussions took the form of dialogues, aimed at letting multiple voices surface.

The first author developed the project idea, invited the participants, and moderated the group discussions. In order to delve deeper into methodologically important aspects of the researcher role in qualitative health research, it was deemed important that all the group participants were anchored in health science and experienced in traditions in which qualitative approaches are highly valued. Representing a diversity of research designs and traditions was also deemed important, as methodological challenges may surface differently in different designs (see Table I).

**Table I:** Overview of research projects, which worked as empirical examples in the reflective group discussions, including methods, traditions, and authors.

Project	Methodological approach	Researcher in the empirical example/author in this article
Empirical example 1: Lived experience of chronic pain and fibromyalgia: Women's stories from daily life	In-depth interviews, phenomenological study	First author (MR)
Empirical example 2: Cancer as a life-changing process: Women's experiences five years after treatment for gynaecological cancer	In-depth interviews, phenomenological study	Third author (RJTS)
Empirical example 3: GPs’ negotiation strategies regarding sick leave for subjective health complaints	Focus-group-interviews, hermeneutic study	Second author (LHM)
Empirical example 4: What do we have to offer? Reflections on the experiences of genetic counselors in Norway	Focus-group-interviews hermeneutic study	Fourth author (ÅL)
Empirical example 5: Datooga, and Dealing with men's spears’: Datooga pastoralists combating male intrusion on female fertility	Participant observation, ethnography	Last author (AB)
Empirical example 6: Utilizing theatrical tools in consultation training. A way to facilitate students’ reflection on action?	Pedagogical study. Health education/drama pedagogics	Fifth author (TJ)

Complete references are included in the list at the end of the article.

In the first group discussion, we openly shared our experiences as researchers in our own projects. No specific themes were introduced by the moderator, but the participants were encouraged to spontaneously bring up themes they considered important. Each participant then chose one research project/empirical example from which she made drawings revealing important topics of her own experience as a researcher. We did not put any restrictions on ourselves as to the drawings, which were meant as a creative way to come up with preliminary discussion themes. The participants worked in pairs to consider at some length what was communicated in the drawings about the researcher role. The crux of the content was later shared and discussed collectively. The discussion was recorded and transcribed. A preliminary analysis of the concrete researcher experiences and meta-reflections from the discussion was performed by the first author. The transcripts and the drawings were circulated to the group participants before the second group discussion, together with preliminary themes and emerging patterns based on the first author's readings of the transcript. Already, at this stage, shifts in the dynamics of the relations between interviewer and interviewee, and researcher vulnerability, were emerging as preliminary themes.

During the second group discussion, based on the preliminary themes deemed most interesting, a decision was reached to deepen our knowledge about the ways in which our own experiences of research-related relations seemed to move us beyond established knowledge of the imbalance in which the researcher holds a privileged position.

Between the second and third group discussion, transcripts from the first two group discussions were read and analysed by first and last authors, looking for concrete examples and meta-reflections that deepened the key issues chosen by the group. The different examples of researcher experiences revolved around more or less explicitly emerging shifts and ambivalence related to knowing and non-knowing positions of the parties in the phase of co-producing the research material. Examples of negotiations, related to whose agenda was directing the production of the research material, emerged in diverse ways. The examples were categorized under headings highlighting the social status of the participants and the researchers, as well as the knowledge positions of the different parties pertaining to the phenomena under study.

During the third group discussion, the analysis was discussed and key issues further developed. This included discussions pertaining to problematizing the notions of “studying up and down” on the basis of the empirical examples. Literature on researcher reflexivity in qualitative health research was familiar to the participants at the outset of the reflexive group discussions, but a fresh literature search was undertaken at this stage, focusing on themes related to the imbalance in the researcher–researched relationship in qualitative research, and researcher vulnerability.

In the fourth group discussion, we once more worked in pairs to develop our meta-reflections around our own experiences as researchers in the six chosen research projects, summing up and discussing the key issues. Notes were taken during this session as well, and a post-group summary was written.

Between the fourth and the fifth group discussion, the participants worked in pairs or separately to write up their researcher experiences, based on the concrete projects. Drafts of textual presentations were sent to the first author, who wrote a comprehensive preliminary paper that was circulated to the participants.

Discussions during the fifth and the sixth group gatherings concerned revision and refinement of the text. The first and the last author had continuous discussions during the writing process.

## The research projects—Differences and common ground

The projects from which the meta-reflections about experiences were drawn were different with regard to aims, research tradition, and research design. However, as stated above, they were all located within the qualitative research tradition of the health sciences, and were epistemologically grounded in the humanistic or social science traditions, as can be seen in Table I.

Two projects (empirical example 1 and 2) were anchored in a phenomenological life-world perspective, and were based on research material produced through in-depth interviews. The projects shared a common interest in exploring phenomena concerning living in “a changed world” related to profound changes in health condition. Both studies involved reflexive practices to create an awareness of the researchers’ pre-understandings. In the first study (Råheim & Håland 2006), women with fibromyalgia were interviewed about living with chronic pain. The second study (Sekse, Råheim, Blaaka, & Gjengedal, 2009; Sekse, Gjengedal, & Råheim, 2012) focused on the experiences of long-term survivors of gynaecological cancer. The researcher–participant relationships in these two projects might be characterized as essentially asymmetrical and “studying down.” Nevertheless, important shifts in who defined the relevant body of knowledge were experienced.

Two studies produced data through focus group interviews (empirical example 3 and 4), research material substantially depending on the interaction within the groups. Both projects aimed to gather knowledge about how to handle challenging cases and ethical dilemmas in professional practice, and they were both anchored in a hermeneutic tradition. One project focused on challenges and problematic aspects of genetic counselling practice (Lunde, Nordin, & Strand, 2014). The second (Nilsen, Werner, Maeland, Eriksen, & Magnussen, 2011) focused on sick-leave decision-making based on general practitioners’ (GPs’) consultations with patients who have complex health issues. In both studies, the researcher was the group moderator. The relationship between researcher and researched in these two studies can be characterized as asymmetrical, such that the asymmetry worked both ways: the researchers held a “superior” position in relation to the participants in terms of planning and leading the project, while the participants/professionals held a “superior” position pertaining to professionally based knowledge within the actual field of research. These studies also actualize studying the privileged, the experts in the field, or “studying up.”

The fifth study (empirical example 5) was a classical field study anchored in ethnography (Blystad, 1999; Blystad & Rekdal, 2004). The researcher lived in a Tanzanian pastoral community for a period of more than 2 years, exploring maternal practices related to pregnancy, childbirth, and infant feeding. The aim was to generate knowledge on the perceptions and practices related to the reproductive process in a community with substantial cultural emphasis on fertility but in a context of extreme marginality and a high prevalence of infant death. Shifting between positions in this project is based on experiences with the participant observer role, a role located at the heart of ethnography. That implied continuous shifts between “inferior,” non-knowledgeable, insider positions and “superior,” knowledgeable outsider positions.

The last study (empirical example 6) was a pedagogical project anchored in the context of health education. A model of group-based communication training for medical students was developed with the help of simulated patients (SP) and theatrical devices. Theoretical perspectives were grounded in pedagogy and in theatre science. In the sub-study referred to below (Jacobsen & Baerheim, 2005), the researcher simulated a particular patient during the training session while medical students acted as the patient's GP. How the students experienced the communication training and what they learnt was evaluated afterwards. The dual role as researcher and SP provides the starting point for reflections on researcher vulnerability from this project.

## Knowledge positions and researcher vulnerability—Shifts and ambivalence

In the following, we will highlight and reflect on shifts related to knowing and not-knowing positions between the researcher and the researched that emerged during discussions. These shifts were intertwined with the power of defining the relevant body of knowledge. In particular, we discuss transitions in terms of who appears to set the agenda or define the terms, and we discuss the vulnerability inherent in the researcher role during the co-production of research material.

### Distracted by illness stories

A prime example of partly losing control of the research agenda from the in-depth interview studies (empirical example 1 and 2) was related to an experience of being diverted by stories of illness. Of interest from the researchers’ points of view was the current experience of living with chronic muscle pain (example 1) and living as a long-term survivor of gynaecological cancer (example 2). The study participants, however, seemed to seize the opportunity to tell their “full” illness stories to someone who had the time to listen, stories that were accompanied by strong emotions. The emotions were vital in this context and made it difficult to interrupt participants. It was unclear whether or not the illness stories had been accepted in the participants’ many encounters with health care workers; for some, their illness stories had been ignored. The context of encounters with health care workers in the actual projects seemed vital. Both researchers were experienced qualitative researchers. Nevertheless, the subjects’ wish to reveal a high level of suffering, and the intensity of the illness stories took the researchers by surprise. The researchers felt ambivalent because the lengthy illness stories occupied more time than had been initially planned. These stories moved the focus of the interviews beyond the research agenda, but the ambiguity about when and how to interrupt the interviewees was experienced as challenging.

This illustrates a particular challenge of participants’ bringing their own agenda into the interviews (Karnieli-Miller et al., 2009). The narrators in these cases talked about what they felt most strongly, including experiences more or less relevant to the study in question. A need to get the illness story off one's chest, finally to be listened to, might indeed have been a factor motivating the patients to take part in the studies. If the researcher is also a health care worker, this knowledge can further fuel the fire of disclosure. The researcher and health care worker roles can become blurred in the research interview situation (Hewitt, 2007; Jack, 2008; Tee & Lathlean, 2004). The participants’ perceptions of the interviewer, including her professional role, can influence the interaction, and hence the information that is revealed (Richards & Emslie, 2000). In one of the projects referred to above, the women who participated did not know about the researcher's professional role as a physiotherapist. In the other, the participants did know that the researcher was also a nurse. The fact that the studies were based in the health care establishment (University Hospital, Faculty of Medicine) might have influenced the participants’ conduct in both studies. Furthermore, the participants could have been motivated to elaborate on the suffering during the interview, as encouragement to reveal personal experiences could have a potential “therapeutic” dimension. Similarities between research interviews and therapeutic encounters have been recognized (Kvale & Brinkmann, 2009). Although therapeutic effects are rarely aimed at by researchers, attentive and empathic listening, and encouraging reflections on what is being expressed might be perceived by the participants as encouragement to narrate detailed tales of illness (Hewitt, 2007; Hutchinson, Wilson, & Wilson, 1994; Lowes & Gill, 2006; Richards & Emslie, 2000).

During the interview stage, the researcher is dependent on the participants’ willingness not only to take part, but also to share their experiences and thoughts about the topics in question (Karnieli-Miller et al., 2009). The researchers considered it important to listen to the illness stories, first and foremost to show respect, but also to gain the trust of the participants, which is essential for a constructive qualitative research encounter. Besides, illness stories might well bring about contextual insights of importance to the understanding of the phenomena to be explored, in our context to the understanding of living with chronic muscle pain or as long-term survivor after cancer. However, including the “full” illness stories had not been planned and it took time away from the key focus of the research.

The balance at play between knowing and non-knowing positions illustrates several points of interest. It is claimed, for instance by Kvale (1996), Brinkmann and Kvale (2005) that the empathic, caring, and empowering atmosphere of equality aimed at in qualitative interviews may conceal power differences and hence be ethically questionable. The researcher's dependence on the trust of participants to get their stories can indicate that the dialogue taking place is used as a strategic instrument that works as a cover for the exercise of research-related power. We have indicated that listening to the illness stories of the research participants was important for establishing mutual trust, which might have been a gateway for accomplishing the researchers’ agendas. As such, listening included a strategic element, which we surely acknowledge is a part of qualitative research interviews. However, being guided by respect and ethically sound reasoning, as well as constantly operating through an open and dwelling attitude, contradicts the notion of attentive listening as “a fake.” Indeed, we will argue that it would have been impossible to gain mutual trust and rich descriptions if the researchers had not been genuinely interested in the experiences of the researched. According to phenomenological methodology, a genuine interest coupled with an attitude of openness and wonder that puts pre-understandings at risk, is essential in order to explore lived experience in any depth (Dahlberg et al., 2008; Van Manen, 1997). However, as we have seen in the cases above, genuine interest and attentive listening also risk paving the way for participants to reveal wells of sensitive information, as well as the risk of moving the interview away from the main research agenda. Difficult ethical choices had to be made during the interview situation. The challenges experienced have some general relevance for the art of in-depth interviewing.

### The inherent researcher vulnerability in In-depth interviews

A common theme in the in-depth interview studies relates to researcher as well as participant vulnerability. Hewitt (2007, p. 1151) underscores that moral questions can arise at any time during in-depth interviews, depending on the types of disclosure, unintended consequences of trust and emotional closeness, as well as varying competence in communication skills and ethically sound reasoning on the part of the researcher. Overly intrusive interviews mean exploitation, and might harm participants (Hammersley & Atkinson, 1983/1992; Kidd & Finleyson, 2006; Richards & Schwartz, 2002).

In the in-depth interview studies considered here, the researchers were involved in stories of great emotional intensity. They were challenged to catch and interpret signs and expressions, tones located beneath and between what was literally communicated in words, in order to make choices about the welfare of the woman/participant. In preparation for the interviews, raised awareness of the importance of not being intrusive was practised. However, both researchers in the in-depth interview studies were acquainted with feelings of guilt, and were touched deeply by the participants’ stories. It is claimed that it is necessary to be deeply absorbed by the participants’ expressions and empathically touched by participants’ to, at least partly, understand what might be at stake in the life-worlds of participants (Angel, 2013), and that such absorption is also paramount to ethically sound research (Malacrida, 2007). However, had we triggered reactions that could add to the women's burden in the long run? If self-disclosure meant re-opening wounds without the opportunity to work them through, it could potentially cause harm. On the other hand, sharing sensitive experiences might invoke relief, self-acknowledgement, and imply a possibility of looking at experiences anew (Hutchinson et al., 1994; Lowes & Gill, 2006). Participants who agree to take part in a study of this kind will, nevertheless, often be unprepared for what they are consenting to and what they may actually reveal (Richards & Schwartz, 2002). The process of qualitative health research is not always predictable for either participants or researchers (Kidd & Finleyson, 2006). Furthermore, what participants communicate just after the interview might later be reversed, adding to the complexity of these issues (Murphy & Dingwall, 2004). As stated by Hewitt (2007, p. 1157), an acknowledgement of the complexities of researcher–researched relationships in in-depth interview studies implies being sensitive to the risks to participants, a continual concern. We fundamentally acknowledge this complexity, and find that enhanced ethical awareness on the part of the researcher is paramount.

Still, we will argue that there is an unsolvable dilemma implicit in in-depth interview studies, where aiming at rich descriptions is a key concern, often implying disclosure of sensitive topics, while at the same time ensuring that one does no harm to participants. We agree with Rager (2005), Lalor et al. (2006), Dunn (1991), Kidd and Finleyson (2006), and Malacrida (2007), who claim that researcher risk vulnerability regarding “compassion stress,” the danger of being emotionally drained. We will add that stress, accompanied by feelings of guilt, is underestimated in qualitative research generally. To explore knowledge about sensitive topics in peoples’ lives entails the “superiority” of the researcher position, but which pertaining to ethically demanding choices and emotional involvement nevertheless implies researcher vulnerability.

### The challenge of hierarchy and status in group interviews with professionals

The participants in the group interviews (empirical examples 3 and 4) were highly qualified health professionals, indicating expert knowledge within the research topics of interest, and holding a superior social role compared to the patients in the in-depth interview studies. The researchers held a privileged position in terms of being the ones who were in charge of the research projects’ agenda. The researchers and the researched also possessed a shared body of knowledge by virtue of having similar or related professional roles. At the same time, a certain “inferiority” in terms of professional knowledge existed between the parties; a relatively newly educated genetic counsellor moderated group interviews with experienced genetic counsellors and geneticists (medical doctors specialized in genetics), and an experienced physiotherapist moderated group interviews with GPs. These studies illustrate research situations in which the challenge of interviewing peers and/or professionals enters the picture, another challenge with methodological implications (Coar & Sim, 2006). The two group interview studies clearly contained an element of “studying up,” moving the researchers into research fields characterized by less “control,” which again is readily related to challenging attitudes among the researched and difficulties of access to information, as noted by Nader (1969/1972, p. 301). The very fact of using group interviews might, moreover, have increased this particular methodological challenge.

The researchers and moderators of the group discussions did feel that the participants questioned their expertise in the field, which primarily emerged as resistance or lack of responsiveness to some of the questions introduced. Furthermore, a hierarchy based on the classical distinction between objective, fact-related knowledge in contrast to knowledge as subjective and experience-related surfaced in both focus group studies. The researchers were interested in learning about how the informants acted and made judgments in specific challenging situations, and this involved asking for the participants’ experience-based knowledge. However, in the group discussions, the researchers and moderators found it challenging to get the participants to describe and reflect on real-life situations experienced in their own practice. Participants quickly turned to responding formally with generalized replies and fact-based knowledge such as health policy, legislation, and so forth.

It should be acknowledged that the topics of discussion in these projects imply medical assessment of substantial complexity, and to present revealing clinical examples may not be easy. Caution related to the disclosure of patient information may add to the challenge. Notwithstanding these points, the potential danger of being exposed and made vulnerable to peers is inherent in revealing subjective experience from one's own practice, a vulnerability that may be experienced as contradictory to the professional role as a doctor, a geneticist, or a genetic counsellor, and might have been important in our context. The research participants, who had expert knowledge about their professional practice (insiders), might, despite confidentiality and anonymity assertions, have felt slightly threatened by a researcher/moderator (outsider) whose intent was to explore politically and clinically potent challenges inherent in their practice. The study participants might legitimately wonder whether the researcher intended to test their professional competence, and/or place their profession in a “bad” light in the professional community. Hesitation to reveal information to colleagues, not just researchers, concerning one's own ways of solving the challenges discussed may also have been a constraint in the group discussions. In Coar and Sim's study (2006), in which both interviewer and interviewed were professionals (GPs), several participants regarded the interview as a test of their professional knowledge. Other studies have also noted that participants and professionals believe that their interests and professional identities are threatened during research (see e.g., Enosh & Ben-Ari, 2010). The perceptions of the researcher and the researched of the research agenda might thus not always be in harmony. Group interviews may also be challenging for the researcher because of the inherent strengths of a group of individuals, who can directly oppose the researcher's agenda.

In the studies with the GPs and genetic counsellors, minor “battles” seemed to be played out, in which the study participants alternated in terms of who was guiding or guarding the knowledge presented, including moments in which the researchers managed to move the discussion in the direction that was desired for the productive generation of knowledge. Neither the researcher in the sick-leave decision-making study (empirical example 3) nor the researcher in the genetic counselling study (empirical example 4) attempted to force the discussions in a preferred direction. Rather, the researchers repeatedly asked for concrete examples in order to gain knowledge beyond the formal, and made continuous attempts to hear participants dwell on the experienced intricacies of actual decision-making processes.

A more comprehensive understanding of the negotiations taking place about the research agenda involves insight into the context at hand and what might be at stake for both the participants and the researcher (Coar & Sim, 2006; Enosh & Ben-Ari, 2010; Vitus, 2008). We have indicated that the participants in both of the focus group discussion studies might have felt that their professional identities were being scrutinized. One cannot be entirely sure that the researchers and the participants were in full agreement about what the research agenda actually implied, although the aims of the research were shared before the discussions. Negotiations and resistance regarding the discussion of problematic clinical cases are, in the research literature, associated with a challenge of revelation. In such cases, the social status of the parties involved may also emerge as significant (Coar & Sim, 2006; Richards & Emslie, 2000). In the focus group studies considered here, the symmetry as well as the asymmetry in the researched–researcher relationship represented a dimension of power that the researchers experienced as challenging and as somewhat unpredictable during the course of the research encounters.

### The child's role: Being at the mercy of the study participants

The next case reveals examples of researcher vulnerability experienced within a classical ethnographic study (empirical example 5). As pointed out, for instance by Hammersley and Atkinson (1983/1992), there is a substantial possibility in ethnography for informants to control the information revealed, not least when studies are carried out in foreign contexts. To illustrate the often shifting character of ethnography, Werner and Schoepfle (1987) have described the participant observer role in fieldwork as a process, starting out with descriptive observation, where taking a child's role is dominant, followed by a more “focused observation” as the cultural knowledge about the field increases, and finally moving into phases of more “selective observation.” Intertwined in such a process are changing relationships between the researcher and the researched.

A classical metaphor for the ethnographic fieldworker is the child who is to be socialized into a particular culture or subculture. As such, the researcher is from the start placed in an “inferior” position pertaining to relevant cultural knowledge. The “innocent” child and ethnographer is simultaneously a conscious and informed researcher working systematically on his or her research agenda. The agenda will be more or less transparent to the study participants, depending on how well a particular research topic can be made sensible in the research setting. An ethnographer's taking on the role of a child has its advantages, especially in the early phases of fieldwork. It allows the ethnographer to pose questions that might appear as uninformed, even naive, and might not be perceived as immediately threatening because they are coming from “a child” who is learning.

Beyond the role of the child, the role of the “insider” is sought within ethnography: being and living among the researched, becoming someone to be trusted and thus allowed access to internal matters. The attempts at gaining mutual trust and reaching a sense of or some degree of closeness to the informants lies at the heart of the ethnographic approach, and depends on considerable time being spent in the field. The “insider” role, however, is continuously articulating with the “outsider” role, which is also inherent in the participant observer role, as the researcher commonly comes from “outside” the studied field.

A particular challenge experienced in the ethnographic study we considered was the study participants’ ways of controlling information, particularly during the early phase of the fieldwork. One area that was perceived as a challenge was that of controlled exclusion: not only the careful sorting of information to be presented to the researcher, but the rigorous denial of access, the distancing or exclusion of the researcher from smaller or larger arenas defined by the researched. Dependent as the ethnographer is on guidance (and possible translation), the potential for control of information passed on to the researcher is more or less limitless, potentially jeopardizing the researcher's project. Despite the fact that the researcher in this project was invited to attend a vast number of relevant events and situations that could provide knowledge about pregnancy and birth-related perceptions and practices, she had, for months, an accompanying feeling of being guided away from core information, and even of being cheated. The experience of being part of a game was not entirely unlike what Angrosino described from his fieldwork (Angrosino & Mays De Pe'rez, 2000):

Even in such a highly circumscribed culture …(referring to his field site), people could experiment with styles of interaction and involve the visitor (researcher) in subtle, yet very revealingly subversive power games, games that inevitably shaped both *what* the ethnographer observed and *how* he interpreted what he saw. (p. 681)

The potential for the researched to control what a researcher is introduced to is obviously fully within the rights of the study participants, and is a principle located at the very core of any research endeavour. Nevertheless, the informants’ ability to control, deploy, and manipulate again raises questions around the notion of the researcher's exclusive power. Diverse forms of participant resistance have been described in the ethnographic literature (Adler & Adler, 2002). Karnieli-Miller et al. (2009, p. 283) refer to the participants’ strategic use of problematic interview behaviour (such as flattery, flirting, and so forth), shift of topics, and even the decision to end the interview or cooperation altogether. On the basis of subtle or overt shifts in power relations between the parties, the awareness of the co-construction of knowledge can become more or less acute. Goodwin, Pope, Mort, and Smith (2003) write:

The community being researched is not a passive component; it also has a bearing on what the researcher is included in and excluded from. The informants were also agents in the shaping of the data, the data-collecting opportunities, and the course of the fieldwork. (p. 576)

At the same time, in the co-construction of knowledge made possible through the symbiotic relationship between researcher and researched lies one of the substantial advantages of ethnography. The closeness will often, with time, generate an openness and permissiveness, which may imply seemingly endless learning opportunities. However, the dependence on the close relationships with the informants simultaneously sheds light on the precariousness and vulnerability not only of the informants, who may have difficulties controlling the information ultimately generated from the research, but the vulnerability of ethnography as a research approach, as well as the vulnerability of the ethnographer in the process of learning.

In the current study, the researcher gradually gained access to more domains, and later fieldwork revealed the immense impact of her own position for the knowledge gained. She was provided with extensive access to the women's ritual reproductive sphere after being married, giving birth, breastfeeding etc. gaining closeness through the sharing of highly praised bodily transitions, a type of access she had not been granted while still “a girl.” The ethnographic experience also emphasized the fundamental importance of developing trust and close relationships. The potential for control of information is obviously particularly extensive at a point when the researcher knows few of her study participants, and when simultaneously the researcher is relatively uninformed about the field of study, that is, during the phase when the researcher's role as “child” is most prominent. The gaining of closeness to the field is thus part of a process of becoming more knowledgeable about culture and context, the handling of language and codes, and of the continuous building of what is often experienced as true friendship. Karnieli-Miller et al. (2009) explain that: “to gain access to the participants’ private and intimate experiences—his or her story—the researcher must enhance a sense of rapport with people and needs to build a considerate and sympathetic relationship and sense of mutual trust” (p. 282). This point pertains to all qualitative research endeavours, but is particularly pertinent in ethnography with its common demands for long-term interaction. In the study, we considered the experience of being gradually more at ease with the continued outsider role, the learning process made the researcher more of an insider. “Interaction is always a tentative process,” Angrosino and Mays De Pérez (2000, p. 683) write, referring to the mutual testing out of the perceptions of one's own and the others’ roles that takes place over time in ethnography. As such, the relationship between researcher and ethnographer, and researched, and hence each person's role toward the other, is not fixed and permanent within ethnography; rather, “their behaviors and expectations of each other are part of a dynamic process that continues to grow throughout the course of single research projects” (ibid p. 683). In a similar vein, we have indicated that the role of researchers as interviewers in the in-depth interview studies and in the focus group discussion studies were not fixed during the course of the interviews. Shifts took place both in relation to definition of the relevant body of knowledge, and the particular position of the researcher in knowledge production. Partly due to the time dimension and the demands of participation, the role of the participant observer is indeed far from static or fixed, but is constantly transformed during the course of the fieldwork (Werner & Schoepfle, 1987).

### The vulnerability in designs with especially demanding inherent dual roles

In the final example, we shed light on how researcher vulnerability seemed to be part and parcel of the dual role of the researcher. In the pedagogical study (empirical example 6), the researcher simultaneously pursued the researcher role and the actor role, portraying a patient during communication training. Two focus group interviews with medical students were conducted after the communication training. The researcher thus shifted from acting the role of a particular patient in front of a group of medical students, to moderating the focus group discussions that evaluated the training from the students’ perspective. The students who participated in the focus groups were either solely a part of the student audience, which was encouraged to comment and suggest “ways to go” in the medical encounter played out in front of them during time-outs led by a teacher and moderator, or they were also involved in the acting as GPs in the simulated encounter.

The character of the SP was a young woman. She was shy, almost nonverbal, someone who gets very easily hurt and starts crying when challenged on personal matters. The topics of the training were “the withdrawn patient” and “breaking bad news” (the patient was told that she has cancer). To portray this patient was demanding, and the actress had to use most of her proficiency and skills as an actor to create a credible character. This created an ambivalent situation; she felt emotionally drained after the performance, and found it difficult to shift from the role of the actress to the role of the researcher who moderated the group interviews. Despite the fact that she was a professional actress and well acquainted with varied responses from audiences, she felt at the mercy of the students’ evaluation in unexpected ways. She found herself wishing for the students’ approval as an actress while simultaneously wanting to be genuinely open to the students’ views of the learning potential of this particular pedagogical practice, with this latter concern demanding the distanced approach of a researcher. Role confusion of both parties could contribute to an unsharpened reflection.

As Malacrida states (2007, pp. 1329–1330), engaging in emotionally challenging research topics and relationships has the potential to unsettle researchers’ well-being, and challenge their self-understanding as researchers. Being in a more emotionally charged research context than initially expected might imply underestimating the strength of the emotional reactions (Dickson-Swift, James, Kippen & Liamputtong, 2008; Rager, 2005). It puts the researcher at risk of becoming emotionally drained (Dunn, 1991; Lalor et al., 2006). To take on the dual role as researcher and SP in the development of this particular pedagogical practice exacerbated the emotional challenge, and made it difficult to find a balance between insider–outsider positions (Burns et al., 2012; Dwyer & Buckle, 2009). Parallels to the vulnerability inherent in the participant observer role in the ethnographic study are present, particularly the feelings of being at the mercy of the participants. The manner in which the researchers opened themselves to exposure placed them in a vulnerable position. In an ethnographic context, the researcher will commonly have a long-lasting relationship with the study participants, which implies opportunities to re-evaluate the course of events and modify ways of approaching demanding topics and situations. This was not the case in the pedagogical project which enhanced the sense of overall vulnerability.

## Concluding remarks

In this article, we have made an attempt to shed light on the researcher–researched relationship in different qualitatively anchored studies carried out within health science. We have concentrated on the phase in which the research material is co-produced by the parties, and the researcher is highly dependent on participants’ knowledge about the phenomena under study, and on their willingness to share. Flyvbjerg, cited in Karnieli-Miller et al. (2009, p. 282) argue that the study of power relations should go beyond the normative level and be anchored in the real practices of qualitative research. In this article, we have anchored our analysis of shifts and ambivalence in the researcher–researched relationship by drawing upon concrete examples from our own research. The four main qualitative approaches represented; the phenomenological in-depth interview studies, the focus group discussion studies, the ethnographic study, and the pedagogical study, held a common aim of diminishing the distance between the researcher and the researched, and creating an anti-authoritative researcher–researched relationship. This meant moving into and confronting complex negotiations about the research agenda, about which knowledge was to be counted as relevant, shifts in “inferior” and “superior” knowledge positions, as well as ethical dilemmas. The scenarios that emerged challenged the researchers partly to re-think the research agenda, but it also rendered them vulnerable to substantial emotional stress. The dual role as insider and outsider, participant and researcher, added to the challenge. “Interaction is always a tentative process that involves the continuous testing by all participants of the conceptions they have to the roles of others,” Angrosino and Mays De Pérez (2000, p. 683) write, with reference to ethnography. Researchers’ and participants’ roles are not fixed, but develop during the projects. The empirical examples in this article indicate that these are points of relevance for qualitative research projects, across designs and traditions.

In order to handle shifts in positions between research parties, shifts which are intertwined with ethical dilemmas, the practice of continuous reflexive awareness is paramount. The same holds true for the context of knowledge production; scrutinizing critically what can be at stake in the encounters between researcher and researched, and one's own role in knowledge production. We argue that sharing and discussing these concerns in research teams and groups, where senior researchers as well as novices meet, should be regular practice. The value of reflexive self-awareness among researchers has been contested. Personal disclosure can fall into an infinite regress of excessive self-analysis at the expense of the research aims (Finley, 2002; Gergen & Gergen, 2000). However, along with Finley (2002, p. 532), we feel that the other pitfall is to avoid reflexivity altogether. Although fraught with ambiguity, a lack of critical awareness about the impact of the research context, perspectives chosen, methodological choices made, and, in this context, the presence of the researcher, might seriously hamper the knowledge claims made. Finally, we support Malacrida (2007, p. 1339) who writes that “reflexive research also should involve emotional care not only for participants but for researchers themselves.”
